# Against the reduction of modality to essence

**DOI:** 10.1007/s11229-017-1667-6

**Published:** 2018-01-05

**Authors:** Nathan Wildman

**Affiliations:** Tilburg Center for Logic, Ethics, and Philosophy of Science (TiLPS), University of Tilburg, PO Box 90153, 5000 LE Tilburg, The Netherlands

**Keywords:** Essence, Modality, Necessity, Possibility, Ontological dependence, Grounding

## Abstract

It is a truth universally acknowledged that a claim of metaphysical modality, in possession of good alethic standing, must be in want of an essentialist foundation. Or at least so say the advocates of the *reductive-essence-first* view (the REF, for short), according to which all (metaphysical) modality is to be reductively defined in terms of essence. Here, I contest this bit of current wisdom. In particular, I offer two puzzles—one concerning the essences of non-compossible, complementary entities, and a second involving entities whose essences are modally ‘loaded’—that together strongly call into question the possibility of reducing modality to essence.

The concept of metaphysical modality has played an important role in the history and development of philosophy; and in no branch of the discipline is its importance more manifest than in metaphysics. In part, this is because modality may be used to characterize what the subject, or at least part of it, is about. For one of the central concerns of metaphysics is how things might have been (and, relatedly, how they must be), and addressing these questions requires appealing to the notion of modality.^1^ In addition, metaphysicians have employed modality to formulate numerous metaphysical claims and to help define a plethora of metaphysical concepts.

A similarly foundational role can be ascribed to the notion of essence. One of the central questions in metaphysics is what things *are*, in the metaphysically significant sense of the phrase. And it is in answer to this question that appeal is naturally made to essence. For example, specifying what Socrates *is* in this metaphysical sense involves explicating his essence, or, at minimum, some of those properties that are essential to him. Further, essence has proven useful in formulating metaphysical claims, and in defining various other metaphysical concepts.

Given the importance to metaphysics of these twin concepts, it is not surprising that philosophers have attempted to get clearer on what they are, and how the two are inter-related. Concerning this latter question, two approaches have been particularly prominent.

The modalist account, which was once ‘so wide-spread that it would be pointless to give references’ (Correia 2005: p. 26),^2^ attempts to reductively analyse or define essence in terms of modality. This is typically done along the following lines:

- **M**       x is essentially F $\hbox {iff}_{\mathrm{df}}$ necessarily, if x exists, then x is F

Recent history, however, has not been kind to modalism. This is primarily due to a series of examples from Fine which purport to undercut the account’s sufficiency.^3^ The most famous of these features Socrates and {Socrates}: necessarily, if Socrates exists, then he is a member of {Socrates}. By **M**, it follows that Socrates is essentially a member of the singleton. But,

...intuitively, this is not so. It is no part of the essence of Socrates to belong to the singleton. ... There is nothing in the nature of a person ... which demands that he belong to this or that set or which even demands that there be any sets. (Fine 1994a: p. 5)

Various attempts have been made to rescue modalism from Fine’s critiques, but these attempts face their own challenges, and the general consensus is that modalism remains ‘fundamentally misguided’ (Fine 1994a: p. 3).^4^

The second, *reductive-essence-first* account (the REF, for short) claims that modalism fails because it gets the story precisely *backwards*: rather than viewing essence as a special case of necessity, the REF treats modality as a special case of essence.^5^ In other words, it attempts to reductively define modality in terms of *essence*, rather than the reverse.

In the past 20-odd years, much work has been done to develop and refine the REF, which has since attracted many contemporary adherents.^6^ And, to be honest, it *is* an attractive position. Essence, conceived of in the REF-manner, looks like a solid foundation for our modal edifice, offering as it does the tantalizing possibility of a fully reductive account of modality.

That said, the aim of this paper is to argue that, at least as it currently stands, the REF fails. This is because (i) one of its core principles is false, and because (ii) there are certain modal facts that cannot be reductively defined in terms of essence.

To demonstrate this, I here present two puzzles. The first concerns entities that, due to conflicting essential properties, are non-compossible. The puzzle is that the essences of these complementary entities generate counter-examples to the REF principle that defines necessity in terms of essence. And, as the most natural ways to avoid or dismiss this puzzle either led to new problems or do not handle all the cases, this puzzle indicates that there is a significant problem with the very heart of the REF.

The second puzzle concerns entities whose essences include certain modal notions. The existence of these ‘modally loaded’ essences suggests that at least some modal facts must be settled prior to settling the essence facts. And, more pressingly, there does not appear to be any viable way to reductively define the modal notions ‘loaded’ inside these essences. Thus the second puzzle is that, in light of these modally loaded essences, a fully reductive definition of all modal notions seems impossible.

Together, these puzzles constitute a direct challenge to the REF, and to the general project of reductively characterizing modality in terms of essence.

The plan is as follows: we begin (Sect. 1) by detailing the various inner workings of the REF, in order to set up the puzzles. This is followed (Sect. 2) by a presentation of the puzzle of complementary entities, and a discussion of various (ultimately unsuccessful) ways REF advocates might to resolve it. The next section (Sect. 3) spells out the puzzle of modally loaded essences, and anticipates several possible responses. Finally, we conclude (Sect. 4) by discussing the wider implications of this paper for the larger matter of trying to understand the relationship between modality and essence.

## Characterizing the REF

The core REF idea is that a ‘metaphysical necessity has its source in the [essences] ... of the objects with which it implicitly deals’ (Fine 2005a: p. 7). From this starting point, we can, according to REF-advocates, provide a complete reduction of modality to essence.

For instance, many necessities can be understood in terms of the *individual essences *of particular entities. For example, ‘Socrates is human’ is necessary because Socrates’ essence includes his being human. Similarly, Socrates is necessarily a member of {Socrates} because it lies in the essence of the set that Socrates is a member, though it does not lie in the essence of Socrates to be a member of the set.

But it isn’t clear that all necessities can be accounted for in terms of individual essences. For example, Socrates’ essence says nothing about the Eiffel Tower, and the Tower’s essence is equally silent about Socrates. So, neither of their individual essences explains their necessary distinctness.

What does account for this necessity is Socrates and the Towers’ *collective essence*—that is, the essence of Socrates and the Tower taken together. Generally, the collective essence of some plurality $\Delta$ includes everything that is essential to each of the plurality’s members, as well as additional things that emerge when we consider them jointly.^7^ Thus it is part of the collective essence of Socrates and the Tower that Socrates is human, the Tower is a tower, and that the two are distinct, despite the fact that neither of their individual essences settles this latter point (Correia 2012: p. 642).

More formally, let ‘’ be read as ‘it is true in virtue of the essence of the *xx*s that P’, where ‘*xx*’ denotes a non-empty plurality of entities, and ‘P’ denotes a sentence. Taking individuals as the limiting case of a plurality, the REF defines necessity like so:

- **NEC**            

Importantly, the quantifier here is taken to range over ‘all possible objects, and not just over the actual’ (Fine 1995a: p. 244).

Then, letting *U* be the universal plurality—the plurality that includes every possible entity—REF proponents use the inter-definability of necessity and possibility to offer a reductive account of possibility:

- **POSS**            

In slogan form: P is (metaphysically) possible whenever the collective essence of all possible things—the ‘Essential Chorus’—does not rule it out.

Hence ‘Socrates is human’ and ‘Socrates and the Eiffel Tower are distinct’ are metaphysically necessary ‘in so far as they are true in virtue of the nature or essence of some objects’ (Correia 2005: p. 15).^8^ Meanwhile, ‘Socrates is snub-nosed’ and ‘Cicero is a farmer’ are metaphysically possible because the universal collective essence does not rule them out. In this way, it seems we can ‘explicate the notions of metaphysical necessity and possibility in terms of essence, rather than vice versa’ (Lowe 2012a: p. 934): every necessity is definable in terms of *some* collective essence, and every possibility is definable in terms of what is not ruled out by the collective essence of every possible entity.

Finally, it is worth noting that the REF is sometimes characterized in terms of *grounding* rather than *defining* modality in terms of essence. Lowe, for example, says that ‘modalities are grounded in essence. That is, all truths about what is metaphysically necessary or possible ... obtain in virtue of the essences of things’ (2012b: p. 110).^9^ This alternative, grounding-based version of the REF replaces **NEC** and **POSS** with:

- **NEC-G**  fully grounds
- **POSS-G**  fully grounds

This grounding-based REF variant offers a slightly different story about the relationship between essence and modality. However, given the standard assumption that grounding is factive, the falsity of **NEC** entails the falsity of **NEC-G**, and the falsity of **POSS** entails the falsity of **POSS**.^10^ Consequently, any counter-examples to **NEC** or **POSS**—like those to be discussed shortly in Sect. 2—will also be counter-examples to the associated grounding principles.

With these matters settled, on to the puzzles.

## The puzzle of complementary entities

Given their acceptance of **NEC**, REF advocates are committed to**NEC**’s right-to-left reading:

- **EN**

And, assuming the factivity of necessity, **EN** entails:

- **EF**

The first puzzle is that *complementary entities*—possible entities that, due to conflicting essential properties, are not compossible—serve as counter-examples to both of the above principles and, consequently, to **NEC**.

The first example of complementary entities concerns events. Suppose we flip a coin c, at time t in location l. There are two possible outcomes—two events that might subsequently occur. One is the event *Tails*, which consists of flipping c at time t in location l and its landing tails. The other is the complement event, *Heads*, which involves c being flipped and landing heads at t in l.^11^

Clearly, both *Tails* and *Heads* are possible—i.e., possibly, c lands heads at time t and in location l, and, possibly, c lands tails at t in l. However, they are mutually exclusive—*Heads* and *Tails* cannot both obtain. Further, if we take events to be *fine-grained* (either because, following Kim (1973, 1976), we take them to be structured, or because, following Lewis (1986), we think fine-grained events are necessary for an adequate account of causation), then part of what makes the event *Tails* the very event that it is, is that it involves c’s landing tails at this time and in this location. Consequently, it is plausible to say that c’s landing tails is partially constitutive of the very identity of *Tails*—it is part of what the event *is*, in the metaphysical sense of the term. Similarly, it is part of what makes *Heads* the very event that it is, that it involves c landing heads at this time, in this location. So, ‘c lands tails at time t and location l’ is true in virtue of the essence of *Tails*, and ‘c lands heads at time t and location l’ is true in virtue of the essence of *Heads*.

This leads to a problem. Given **EF**, it follows that both ‘c lands tails at t in l’ and ‘c lands heads at t in l’ are true. But it isn’t possible for *both* to be true—that would be a contradiction. Similarly, given **EN**, both are necessary. But this is false—after all, both *Heads* and *Tails* are *possible* outcomes of our flipping the coin! And note that, like with **NEC**, the quantification in **EN** and **EF** ranges over all possible entities. So the fact that (e.g.,) *Heads* doesn’t *actually* obtain (because *Tails* actually did) is irrelevant—all that is necessary to generate the problem is the fact that both complementary events are *possible*.^12^

A second case involves tropes. Take this ball b’s redness-all-over-at-time-t trope *r*, and the distinct, mutually exclusive, albeit equally possible, trope of b’s greenness-all-over-at-time-t *g*. Part of what makes the former the thing that it is, is that it involves b’s being red all over at t. Similarly, part of what makes the latter the thing that it is, is that it involves b’s being green all over at t. So, if anything is true in virtue of the essence of r, it is that b is red all over at t. And if anything is true in virtue of the essence of g, it is that b is green all over at t.

By **EF**, these entail that the ball is red all over and green all over at t. But this is impossible. Further, by **EN**, it follows that necessarily, the ball is red all over at t and necessarily, the ball is green all over at t. But both of these necessity claims are false, because, given the possible existence of r and g, possibly the ball is green, and not red, all over at t, and possibly the ball is red, and not green, at t.

Consequently, complementary entities generate counter-examples to **EF**, **EN** and, by extension, **NEC**.

REF advocates might try to circumvent this puzzle by denying that there are any complementary entities. This, however, seems implausible. For one, it is clear that, if we understand events as fine-grained, then certain possible events will be mutually exclusive. Similarly, the identity conditions for tropes naturally deliver complementary cases.

Of course, some might not include events and tropes in their ontologies. But even this won’t block the puzzle, since there are complementary *objects*. For example, Fine himself (1995a: p. 244) suggests that certain conceptions of God and Satan are plausibly understood as complementary—e.g., if it is part of God’s essence that She is more powerful than every other entity, and part of Satan’s essence that he is more powerful than every other entity, then the two cannot co-exist. And the broader essence literature offers other cases. Following Salmon (1981: p. 210), by virtue of their being radically different table-types, Richard, a folding card table, and Judy, a dining table with a removable spacer, both of which could have been made from some specific collection of planks, are distinct. Assuming origin essentialism, Richard and Judy are complementary: if we make Judy, we ‘use up’ the planks, thereby preventing ‘further table productions from those [planks]’ (Rohrbaugh and deRossett 2006: p. 382). In particular, we are prevented from producing Richard.^13^ So there definitely seem to be complementary entities, including complementary events, tropes, and objects.

A second, more plausible response involves conditionalizing. As the problem cases all involve non-necessary entities, mimicking moves used in the debate about so-called ‘weak’ necessities,^14^ a REF-er might modify **EN** and **EF** by conditionalizing upon the *existence* of the relevant entities:

- EN*  (the *xx*’s exist/obtain $\rightarrow \hbox {P}$)
- EF*  (the *xx*’s exist/obtain $\rightarrow \hbox {P}$)

These alternative principles avoid the problematic results described above: the truth of both ‘if *Tails* obtains, then c lands tails’ and ‘if *Heads* obtains, then c lands tails’ is not contradictory, nor is there a problem with both conditionals being necessary. And whenever the relevant entity exists, the associated proposition will be true. Thus, if it is the case that *Heads* obtains, then c lands heads.

Unfortunately, this brings more trouble than it is worth.^15^ Adopting EN* and EF* requires modifying **NEC** to something like:

- NEC* (the *xx*’s exist/obtain $\rightarrow$ P)

However, NEC* only delivers conditionalized necessities, and many of the necessities we want to derive are non-conditional. For example, NEC* only guarantees that necessarily, if Socrates and the Eiffel Tower exist, then they are distinct. Yet the non-conditional necessity is the case too—i.e., necessarily, Socrates is distinct from the Tower.

REF advocates might try to dodge this problem by treating Socrates and the Tower as *essential existents*—i.e., entities whose essences include their own existence. As essential existents, their essences could then guarantee the necessity of the conditional’s antecedent, and hence, by distribution, the necessity of the consequent. In this way, we seem to be able to derive the non-conditional necessity from the conditional. However, it is implausible that Socrates and the Tower are essential existents; indeed, Fine himself says that ‘we do not want to say that [Socrates] essentially exists’ (1994a: p. 6). Further, this kind of solution is going to generate new problems: for example, necessarily, *Heads* is distinct from *Tails*, but we can’t stipulate that these complementary entities are both essential existents without reviving the problems discussed above.

The biggest problem is that it is unclear how to derive an analogue of **POSS** from NEC*. Following the previous method, we might try via a universal plurality *U* and the inter-definability of necessity and possibility: Suppose . Given NEC*, this entails (U exists ). Via inter-definability, we then get (*U* exists ). This is equivalent to  (*U* exists & P). Distribution then gives us .

However, it is *not* possible that *U* exists: *U* is the universal plurality, meaning it includes all the entities, including numerous non-compossible entities (namely, all the complementary ones). Consequently, there is no world where *U*, the vast, contradictory collection of every possible entity, exists. So, the step before distribution fails, and we can’t get an account of possibilities from NEC*.

One might try to avoid this problem by using the collective essences of maximally consistent sub-collections of *U*, but this leads to new difficulties. Suppose that *MC1* and *MC2 *are distinct maximally consistent sub-collections of possible entities, such that the former includes trope g and the latter trope r. There will be much that *MC1* is silent about. In particular, *MC1 *will say nothing about whether, if r exists, it is *not* distinct from {r}.^16^ But (r exists $\rightarrow$ {r}) in turn entails that (r exists $\rightarrow$ r is not distinct from {r}), which is flatly false: necessarily, if r exists, then r is distinct from {r}! And what goes for *MC1* will also go, *mutatis mutandis*, for *MC2*, or any other maximally consistent sub-collection: each of them will deliver false possibilities concerning the entities they do not include. Thus shifting to EN* and EF* does not help resolve the puzzle.

A third and final response REF advocates might pursue is to directly contest the essentialist statements used to generate the counter-examples. Consider the *Tails*/*Heads* case again. What led to the problem was the idea that part of what makes *Tails* the very event that it is, is its involving c’s landing tails. Similarly for *Heads*—part of what makes it the event it is, is that it involves c’s landing heads. Consequently, ‘c lands tails’ looks to be true in virtue of the nature of the former, and ‘c lands heads’ true in virtue of the latter.

However, one might contend that these are *not* true in virtue of the relevant natures. Rather, what *is* true are certain identity statements. For (according to this response) what it is to be *Tails* is to be the event of c’s landing tails at time t in location l, and what it is to be *Heads* is to be the event of c’s landing heads at time t in location l. So what is true in virtue of *Tails’* nature is that *Tails* is the event of coin c’s landing tails at t in l. And what is true in virtue of the nature of *Heads* is that *Heads* is the event of c’s landing heads at t in l. These identity statements do not contradict—meaning no problem for **EF**—and both of their necessitations seem true—meaning no problem for **EN**.

Similar reasoning applies in the trope case: what is true according to r’s nature is that r is the trope of ball b’s being red all over at time t, and what is true according to g’s nature is that g is the trope of ball b’s being green all over at time t. Again, these do not contradict, nor are their necessitations false.

An initial counter is that the embedded definite descriptions are *existence entailing*—i.e., if ‘*Tails* is the event of c’s landing tails at t in l’ is true, then there exists an event that is such that c lands tails at t in l. And, of course, the existence of an event that is such that c lands tails entails that c lands tails. Consequently, by **EF**, it being true in virtue of the nature of *Tails* that *Tails* is the event of c landing tails at t in l entails that c lands tails at t in l. And its’ being true in virtue of the nature of *Heads* that *Heads* is the event of c landing heads at t in l entails that c lands heads at t in l. So, we hit the contradiction. Further, the existence entailing nature of the definite description means that necessity of ‘*Tails* is the event of c’s landing tails at t in l’—as guaranteed by **EN**—entails the necessity of ‘c lands tails at t in l’. And as the same will hold for *Heads’* essentially being the event of c’s landing heads at t in l’, the original problem for **EN** will also emerge.

Of course, not everyone agrees that definite descriptions are existence entailing. With that in mind, a second issue is that this response leads to a kind of ‘revenge’ version of the problem. Consider the fact that *Tails* occurs and the complementary fact that *Heads* occurs—i.e., [*Tails* occurs] and [*Heads* occurs]. Plausibly, it is true in virtue of the nature of [*Tails* occurs] that c lands tails—after all, c’s landing tails is a vital part of what makes this fact the very fact that it is, serving to individuate and characterize it. For similar reasons, it is true in virtue of the nature of [*Heads* occurs] that c lands heads. As both facts are possible, they generate counter-examples to **EF**, **EN**, and **NEC**, even in light of the above response.

REF advocates might try to block this revenge problem by making some distinctions at the level of essence statements.^17^ Following Correia (2006, 2013), let us distinguish between *objectual*, *generic, *and*alethic *essence statements.^18^ The key difference between these is that the first describes the natures of *things*, the second the natures of *ways of being*, and the third of *things being a certain way*. For example, consider:

1. It is essential to Socrates to be human
2. It is essential to being a human to be a rational animal
3. It is essential to Socrates’ being a human that he be a rational animal

Here, (1) expresses objectual essence—it tells us something about Socrates’ nature—while (2) expresses generic essence—it tells us something about the nature of being a human—and (3) expresses alethic essence, as it says something about the nature of Socrates’ being a certain way (namely, his being a human).

Importantly, according to Correia (2013: p. 277),^19^ alethic essence statements cannot be reduced to objectual essence statements. Plausibly, part of what [*Tails* obtains] is, is that [*Tails* obtains] is a fact. However, it does not seem part of what it is for *Tails’* obtaining to be the case is that [*Tails’* obtains] is a fact, nor that there are facts at all. So there is a difference between the former, objectual essence claim, and the latter, alethic essence claim.

Using this distinction, the REF advocate can reject the key claim in the ‘revenge’ argument. In other words, according to this reply, while it is part of nature of *Tails’* obtaining that c lands tails, it is *not* part of the (objectual) nature of [*Tails* obtains] that c lands tails at t in l. So ‘c lands tails at t in l’ is *not* true in virtue of the nature of [*Tails* obtains], and the counter-example does not get going. Instead, the closest thing that is true in virtue of the nature of [*Tails* obtains] is another identity claim: namely, that [*Tails* obtains] is the fact that c lands tails at t in l. But, as before, this does not generate a problem (assuming we set-aside the matter of whether the embedded definite description is existence entailing).

I must confess that I find denying that it is true in virtue of the (objectual) essence of [*Tails* obtains] that c lands tails at t in l counter-intuitive. And, obviously, if this is true, then the above attempt to circumvent the puzzle fails. That said, even if we grant that it is not part of the essence of [Tails obtains] that c lands tails at t in l, we can still get the puzzle going.

Recall again that there are complementary objects: Fine’s example of God and Satan, and the two tables, Richard and Judy, both of which essentially originate from some specific collection of planks. The counter-examples to **EF** and **EN** feature objectual essentialist claims. For example, ‘it is essential to God to be more powerful than any other being’ is a statement of objectual essence, as is ‘it is essential to Satan to be more powerful than any other being’. Plugging these *objectual* essence claims into **EF** gives us a contradiction: God is more powerful than Satan, but Satan is more powerful than God. Similarly, plugging them into **EN** gives us conflicting (and false!) necessities: necessarily, God is more powerful and necessarily, Satan is more powerful. The alethic/objectual distinction does not seem to help in these cases. Consequently, the problem remains.

The general upshot is that complementary entities pose a problem for the REF, in that they entail the falsity of **NEC**. And until a proposal is put forward that handles these cases, the REF is in trouble.

## The puzzle of modally loaded essences

The second puzzle emerges due to the fact that some entities have ‘modally loaded’ essences. Take a particular grain of salt, s. Assuming s is essentially a grain of salt, then part of s’s essence is that s has the potential to dissolve in water. Similarly, if electron e is essentially an electron, then it is part of e’s essence that it has the potential to attract positively charged particles.

Obviously, these potentialities are modal in nature, though there is some debate about how best to characterize them. Some treat potentialities as single or even multiple counter-factual conditionals, while others take them to be a sui generis modality.^20^ However, following Vetter (2011, 2013, 2014, 2015), I contend that we should treat potentialities as *localized possibilities*—i.e., a property of an individual object that expresses a possibility. So understood, ‘x has the potential to F’ is just another way of expressing the claim that ‘x *can* F’, where the ‘can’ expresses metaphysical possibility.^21^ In turn, potentiality-loaded essences just are possibility-loaded essences—that is, it is part of s’s essence that s *possibly* dissolves in water, and e’s essence that e *possibly* attracts positively charged particles.^22^

Now, if it is part of s’s essence that s possibly dissolves in water, then the modal fact that s possibly dissolves in water is partially constitutive of s’s essence. But if this possibility fact is partially constitutive of the essence, then there is a sense in which it is *prior* to the essence of s: speaking metaphorically, we need the possibility fact to already be around before we can ‘build’ s’s essence—it can’t come about after the essence does. We need the modality before we can get the essence.

In short, the existence of these modally loaded essences suggests that at least *some* modal facts are fixed *before* the essence facts are fixed. Some modal notions—e.g. the possibility that is part of s’s essentially possibly dissolving in water—are *prior* to the essence facts.

As well as turning the REF’s core priority thesis on its head, this calls into question the REF’s reductive aspirations: if some modal facts are ‘prior’ to essence facts, then the former cannot be reduced to the latter. For reductive definition is meant to give us a way to account for, or characterize, derivative, less fundamental things in terms of the more fundamental ones. Yet the fact that some modal stuff is needed to ‘construct’ certain essences indicates that at least *this* modal stuff is more fundamental than essences.

This problem is even more pronounced assuming the REF grounding variant. This view says all modality is, one way or another, grounded in essence. As s’s possibly dissolving in water is partially constitutive of s’s essence, the former is a partial ground for the latter. Since partial grounding is irreflexive, this entails that s’s essence is not a partial ground for the possibility. However, **POSS-G** entails that s’s essence is a partial ground for the possibility. But this violates irreflexivity.

The upshot is that the existence of modally loaded essences appears to show that at least some modality cannot be reductively understood in terms of essence.

An initial response is to deny that there are any modally loaded essences. For example, one could argue that essence ought to be restricted to purely *categorical* (i.e., non-dispositional) properties. However, this delivers an impoverished account of essence, and so is unsatisfying.

A *prima facie* better option is to claim that the loaded property is not part of an individual, but rather a collective essence.^23^ So, for example, it isn’t part of s’s individual essence that s has the potential to dissolve in water; rather, it is part of the collective essence s and water taken together. This could then be bolstered by claiming that the loaded properties are essential, but only *derivatively*: for every modally-loaded essence fact, there’s a deeper, modality-free essence fact we can cite to explain it, thereby ensuring that there’s never a modal notion that isn’t accounted for by some essence. For example, while s essentially possibly dissolves in water, this is grounded in s’s essentially having the chemical structure that it does.

Though I have my doubts about whether this response can work for all possibility loaded essences (likely, this will hinge upon whether all potentialities can be reduced to their categorical bases),^24^ even if it were to succeed, the puzzle would still emerge. This is because as well as possibility loaded essences, there are also *necessity* loaded essences: for example, returning to our earlier example, it is plausibly part of God’s essence that She is *necessarily* omniscient, *necessarily* omnibenevolent, and a *necessary *existent.^25^

These necessity-loaded essences generate the same problems as potentiality-loaded essences: if it is part of God’s essence that She is necessarily omniscient, then, because the relevant necessity partially constitutes God’s essence, the necessity is *prior* to the essence; similarly, if Her necessary omnibenevolence is partially constitutive of, and hence partial grounds for, God’s essence, then, *contra* **NEC-G**, God’s essence cannot be a partial grounds for Her necessary omnibenevolence. But, more importantly, there is no obvious non-modal essential property of God that we could cite to explain Her essential necessary omni-benevolence, nor is there any suitable collective essence to foist it upon. So, even if we grant that the above response takes care of possibility-loaded essences, puzzling cases still remain.

Finally, REF advocates could argue that the puzzle turns on too strong a test for non-circularity.^26^ In particular, the puzzle emerges because there are specific *instances* of the analysis that contain facts involving the notion to be analysed—i.e., specific essentialist facts that include modal notions. But it isn’t generally a problem for an analysis to feature such instances. Consider a reductive definition of knowledge in terms of X. Clearly, some of the knowledge to be analysed includes knowledge about what is known. This means there are some knowledge facts which play a role in fixing some of the X facts. Consequently, some knowledge facts are ‘prior’ to X facts. But no one is going to reject the possibility of reductively analysing knowledge in term of X simply because we sometimes need to cite some funny knowledge facts to explain specific X facts.

Similarly, the existence of knowledge about knowledge entails that some knowledge facts are at least partial grounds for some X facts, meaning *these* X facts cannot be partial grounds for all knowledge facts. But this doesn’t undercut the claim that all knowledge facts are (at least) partially grounded in X facts.

More generally, what *would* be evidence of circularity is if the general schema expressed by the analysis (or the grounding claim) contains the notion to be analysed. Yet nothing said above seems to show this. Hence the puzzle is fundamentally mistaken.

This is a good objection, in part, at least for its role in clarifying the nature of the puzzle. For simplicity, take the JTB analysis of knowledge. The ‘problem’ is what to do with knowledge about knowledge facts, like knower k’s knowing that they know that P. Thankfully, there’s an obvious and straightforward story: this iterated knowledge claim can be iteratively analysed, such that the above fact can be analysed as k’s having justified true belief that they have justified true belief that P. In other words, by simply iteratively applying the analysis, we can eliminate the embedded knowledge operator and derive something only using the terms of the analysis.

But try doing the same process with s’s essentially possibly dissolving. The initial, problematic claim is that it is true in virtue of the nature of s that possibly, s dissolves in water. Or, in our notation:

(i)
( (s dissolves in water))

The next step, which should give us a REF-friendly analysis of (i), is to apply **POSS** to the embedded possibility. Doing so results in:

(ii)
( (s dissolves in water)).^27^

However, by making the collective essence of U part of s’s essence, (ii) makes it such that every possible entity is part of the essence of s. Thus, paraphrasing Fine (1994a: p. 6), ‘O happy metaphysician! For in discovering the nature of one thing, he thereby discovers all things’. Further, if we follow Fine (1995b: p. 275) and Koslicki (2012: p. 190) in taking x to depend upon y if y is a constituent of an essential property of x, then this line makes s ontologically dependent upon every possible entity.

For these reasons, (ii) cannot be the right analysis of (i). But the problem is that there does not seem to be any analysis in the offing. Thus the problem: potentiality-loaded essences seem to be counter-examples to the REF’s reductive project.

Similarly, consider God’s essentially necessarily being omnibenevolent. The initial, problematic claim is that it is true in virtue of the nature of God that necessarily, She is omnibenevolent. In the notation, this is:

(iii)
( (God is omnibenevolent))

Now, to get a REF-friendly analysis of (iii), we should apply NEC, which results in:

(iv)
( (God is omnibenevolent))^28^

But this is wrong. God’s essence is very striking, but even it does not feature this sort of essential iteration—that is, it isn’t part of God’s essence that it is part of God’s essence that She’s anything at all.

So (iv) isn’t the REF-friendly account of (iii). And, as before, the problem is that there is no obvious, REF acceptable way to understand (iii). Consequently, necessity-loaded essences like God’s seem to indicate that there are particular modal notions that cannot be reductively analysed in terms of essence.

This, then, is the second puzzle: there is no obvious way to reductively analyse the modalities that appear inside certain modally-loaded essences. Consequently, it appears that the REF fails to completely reductively define *all* modal notions in terms of essence.

## Conclusion

Let’s review. Here, we’ve offered two puzzles, which raise distinct problems for the REF. The first puzzle concerned complementary entities. The issue is that the essences of these entities appear to entail the falsity of **NEC**. And the most natural ways to avoid or dismiss the puzzle either led to new problems or did not handle all the cases. Meanwhile, the second puzzle concerned modally loaded essences. Here, the problem is that these essences suggest that there are some modal facts that must be settled prior to the essence facts. And, more worryingly, there does not seem to be any way to reductively define the ‘loaded’ modalities.

In this way, the former puzzle entails that one of the REF’s cornerstone principles is false, the latter that it’s reductive ambitions are doomed to failure. Together, these results suggest that the REF should be rejected. Given that a large number of contemporary metaphysicians seem to have bought in, this is a fairly significant result. But if we do give up on the REF, where do we go from here?

As far as I can see, there are three options. First, we can take the above as an extended argument for a return to modalism. Of course, given the various problems facing it, I imagine that few, if any, will pursue this route.

Second, we might dig in our heels and attempt to defend the REF. This looks appealing, in part because the puzzles are *puzzles*—they point out troubling oddities that need to be corrected if the REF project is to succeed. And nothing I have said here shows that they are impossible to solve (only that a number of particular strategies for doing so fail). That said, until such solutions are offered, the above puzzles do constitute significant problems for the REF.

Finally, we might give up on any reductive aspirations and, following Hale (2013) and Jubien (2009), claim that, though the two are intimately linked, there is no way to *reductively* define essence and modality in terms of each other.^29^ Obviously, this non-reductive account is less ideologically parsimonious than either reductive options, but perhaps the lesson is that this is the only way forward.

Resolving this debate is beyond the scope of this paper. That said, I hope that the pair of puzzles given here should at least make us wary of the possibility of reductively analysing modality in terms of essence.^30^

## Footnotes

1 Unless otherwise specified, the notion of modality invoked throughout the paper is metaphysical, rather than e.g. conceptual, logical, nomic, or normative. See Fine (2002), Hale (1996, 1999, 2002, 2012, 2013), and Kment (2014) for more on the varieties of modality.

2 That said, see e.g. Marcus (1967), Kripke (1980, fn57) and Plantinga (1974: p. 59).

3
Dunn (1990) raises similar objections, while Torza (2015) offers a refined version of them.

4 See e.g. Zalta (2006), Cowling (2013), Wildman (2013), Brogaard and Salerno (2013), Denby (2014), and Livingstone-Banks (2017); for critical discussion, see Skiles (2015), Wildman (2016), and Steward (2015).

5 Essence can here be understood as primitive (Fine 1994a, b, 1995a, b), or in non-modal terms (e.g. via generalized identity, as in Correia and Skiles 2017).

6 For example, see Fine (1994a, b, 1995a, b, 2000, 2015), Correia (2006, 2007, 2012, 2013), Lowe (2008, 2012a), Oderberg (2007), and Shalkowski (2008).

7 Hence the notion of collective essence in play here is governed by:

*Collectivity*: If *x* is a member of the plurality *yy*, then it is essential to *x* that *P* only if it is essential to *yy* that *P*

Fine (1994b, 1995a) distinguishes between *reducible* and *irreducible* collective essential truths; *Collectivity* flattens this distinction. Note that, in the following, I will occasionally talk as if pluralities are singular entities, and refer to their elements as members; this is purely stylistic, and should not be taken to imply that plural quantification is disguised talk of set-like entities that are something over-and-above the singular objects plurally quantified over. Similarly, I’ll occasionally speak of a collective essence as a singular entity (e.g. ‘the collective essence of Socrates and the Tower’); again, this is stylistic, and should not be taken to imply that there is some entity—the plurality of Socrates and the Tower—whose individual essence I am referring to.

8 Rosen (2010: p. 121) and Fine (2002: p. 265) say something similar. However, Fine (2015) regiments essence statements with a variable-binding essentialist arrow, while Correia (2006, 2013) allows the essentialist operator to be indexed to items of other syntactic categories, such as predicates and sentences. Finally, while this presentation roughly aligns with Fine (1995a, b: p. 247), it slightly differs from Fine (1994a, b), as the latter identifies the metaphysically necessary truths as those propositions ‘true in virtue of the nature of all objects whatever’—i.e.:

- **NEC-UNI**            

However, this is equivalent to **NEC**: if it is essential to *U* that P, then, by existential generalization, there is some plurality *xx* such that it is essential to *xx* that P. Moreover, suppose that for some plurality *aa*, it is essential to *aa* that P. Given that every plurality belongs to *U*, it follows that*aa* belongs to *U*, and hence by *Collectivity* that it is essential to *U* that P. See Michels (ms) for further discussion about how these various definitions are equivalent.

9
Kment (2014), Rosen (2010), and Tahko (2015: pp. 156, 165) appear to favour this grounding version of the REF, as did Leibniz [at least according to Newlands (2013)].

10 Assume **NEC** is false. Then one of the two sides of **NEC-G** is false. Consequently, the corresponding grounding claim must also be false. Similarly for **POSS** and **POSS-G** is false. Finally, if we assume Rosen’s *Grounding-Reduction Link* (2010: pp. 122–6), then **NEC** entails **NEC-G** and **POSS** entails **POSS-G**.

11 Livingstone-Banks (ms) employs a similar example, though in a slightly different context.

12 REFers might try to avoid this problem by not using possibilist quantification, but this walks squarely into the teeth of the problems detailed by Teitel (2017). As such, it is not a viable way for them to go.

13 A slight complication here is the possibility of ‘recycling cases’—*aka* Ship of Theseus cases—where the original material is subsequently recycled and used in a second round of production. This suggests that Richard and Judy aren’t strictly complementary, since we could e.g. first build Judy and then re-use the same materials to build Richard. However, many origin essentialists—most notably Forbes (2002: p. 328)—suggest that origin essentialism entails ‘predecessor essentialism’, according to which exactly where in a recycling sequence a given entity occurs is essential to it. So, taking Richard to be the *first* folding card table and Judy the *first* dining table with removable spacer we could construct from some collection of planks, Richard and Judy are complementary entities. For further discussion on origin and predecessor essentialism, see (Wildman ms-a) and Mackie (2006).

14 See e.g. Kripke (1971), Wiggins (1976), Davies (1981), Fine (2005b), McLeod (2008), and (Wildman ms-b) for more about weak necessity.

15 The following problems also apply to responses that only conditionalize the problematic claims, or conditionalize inside the essentialist operator (i.e., (the *xx* exist/obtain $\rightarrow \hbox {P}$) (the *xx*’s exist/obtain $\rightarrow \hbox {P}$)).

16 This is for broadly the same reasons why Socrates’ essence alone couldn’t account for his being necessarily distinct from the Eiffel Tower: there’s nothing in the collective essence of *MC1* that determines the (non)identities of r and {r}—*MC1* is ‘silent’ on such matters.

17 Thanks to Alex Skiles for discussing here.

18 Fine (2012, fn24) says there is ‘something to be said’ for alethic essence, and Carnino (2014) makes use of the notion to discuss the relation between essence and grounding. Finally, Correia and Skiles (2017) use ‘factual’ rather than ‘alethic’, but employ the same notion to discuss essence, grounding, and generalized identity.

19
Correia (2006) also offers a sustained argument that generic essence cannot be reduced to objectual essence, but we can set this matter aside here.

20 For further discussion, see e.g. Lewis (1997), Choi (2006), Steinberg (2010), Manley and Wasserman (2007, 2008), and Mumford and Anjum (2011a, b, 2014).

21
Vetter (2015) employs this understanding of potentiality to develop an account of modality overall. However, it is unclear whether this larger project succeeds—see e.g. Leech (2017) for more discussion. Thankfully, her characterization of potentiality is independent from this project. Further, one might be worried about equating agency claims with ‘can’ claims (c.f. Spencer 2017). However, as the above examples do not feature agency, I need not take a stand on this matter.

22 Note that the non-modalized property of *dissolving* is not essential to s, since s might exist without ever dissolving (e.g. because it never comes in contact with any water, or because, when we drop it in some water, a wizard casts a non-dissolving spell on it).

23 Thanks to an anonymous referee for pressing me on this response.

24 For further discussion of the possibility of ‘bare’ dispositions, see e.g. McKitrick (2003) and Mumford (2006).

25 This ‘strong’ conception of God is distinct from a ‘little-g’ god whose essence merely includes the non-necessitated properties of omniscience, omnipotence, and existence.

26 Thanks to Alex Skiles for suggesting this line of response.

27 In words: it is true in virtue of the nature of s that it is not true in virtue of the nature of the universal collective essence *U* that it is not the case that s dissolves in water.

28 In words: it is true in virtue of the nature of God that it is true in virtue of the nature of God that God is omnibenevolent.

29 Note that this view leaves open the possibility that the two could be reductively understood in terms of something else entirely (e.g. modality in terms of possible worlds and essence in terms of grounding). See Turner (2010) for some convincing objections to Jubien’s particular project.

30 Thanks to Neil McDonnell, Benjamin Schnieder, Alex Skiles, Richard Woodward, and two anonymous referees for helpful comments/discussion, as well as to the audience at the Grounding, Essence, and Modality Conference in Helsinki. Special thanks to Amanda Cawston for her help and support, and to Bob Hale, whose work on the source of modality has been a continual inspiration. This research was partially supported by the Swiss National Science Foundation sinergia project, ‘Grounding: Metaphysics, Science, and Logic’ (Project 147685).

## References

[CR1] Brogaard B, Salerno J (2013). Remarks on counterpossibles. Synthese.

[CR2] Carnino P (2014). On the reduction of grounding to essence. Studia Philosophica Estonica.

[CR3] Choi S (2006). The simple versus reformed conditional analysis of dispositions. Synthese.

[CR4] Correia F (2005). Existential dependence and cognate notions.

[CR5] Correia F (2006). Generic essence, objectual essence, and modality. Noûs.

[CR6] Correia F (2007). (Finean) essence and (priorean) modality. Dialectica.

[CR7] Correia F (2012). On the reduction of necessity to essence. Philosophy and Phenomenological Research.

[CR8] Correia F, Hoeltje M, Schnieder B, Steinberg A (2013). Metaphysical grounds and essence. Varieties of dependence.

[CR9] Correia, F., & Skiles, A. (2017). Grounding, essence, and identity. *Philosophy and Phenomenological Research*. 10.1111/phpr.12468.

[CR10] Cowling S (2013). The modal view of essence. Canadian Journal of Philosophy.

[CR11] Davies M (1981). Meaning, quantification, necessity: Themes in philosophical logic.

[CR12] Denby DA, Francescotti R (2014). Essence and intrinsicality. Companion to intrinsic properties.

[CR13] Dunn JM (1990). Relevant predication 2: Intrinsic properties and internal relations. Philosophical Studies.

[CR14] Fine K (1994). Essence and modality. Philosophical Perspectives.

[CR15] Fine K, Sinnott-Armstrong W, Raffman D, Asher N (1994). Senses of essence. Modality, morality and belief. Essays in honor of Ruth Barcan Marcus.

[CR16] Fine K (1995). The logic of essence. Journal of Philosophical Logic.

[CR17] Fine K (1995). Ontological dependence. Proceedings of the Aristotelian Society.

[CR18] Fine K (2000). Semantics for the logic of essence. Journal of Philosophical Logic.

[CR19] Fine K, Gendler TS, Hawthorne J (2002). Varieties of necessity. Conceivability and possibility.

[CR20] Fine K (2005). Modality and tense.

[CR21] Fine, K. (2005b) Necessity and non-existence. In his *Modality and tense* (pp. 321–353). Oxford: Oxford University Press.

[CR22] Fine K, Correia F, Schnieder B (2012). Guide to ground. Metaphysical grounding.

[CR23] Fine K (2015). Unified foundations for essence and ground. Journal of the American Philosophical Association.

[CR24] Forbes G, Bottani A, Giaretta P, Carrara M (2002). Origins and identities. Individuals, essence and identity.

[CR25] Hale B (1996). Absolute necessities. Philosophical Perspectives.

[CR26] Hale B (1999). On some arguments for the necessity of necessity. Mind.

[CR27] Hale B (2002). The source of necessity. Noûs.

[CR28] Hale B (2012). What is absolute necessity?. Philosophia Scientae.

[CR29] Hale B (2013). Necessary beings: An essay on ontology, modality, and the relations between them.

[CR30] Jubien M (2009). Possibility.

[CR31] Kim J (1973). Causation, nomic subsumption, and the concept of event. Journal of Philosophy.

[CR32] Kim, J. (1976). Events as property exemplifications. In M. Brand & D. Walton (Eds.), *Action theory* (pp. 159–177). Dordrecht: Reidel. (Reprinted from *Supervenience and mind*, by J. Kim, Ed., 1993, Cambridge: Cambridge University Press.

[CR33] Kment B (2014). Modality and explanatory reasoning.

[CR34] Koslicki K, Correia F, Schnieder B (2012). Varieties of ontological dependence. Metaphysical grounding: Understanding the structure of reality.

[CR35] Kripke S, Munitz M (1971). Identity and necessity. Identity and individuation.

[CR36] Kripke S (1980). Naming and necessity.

[CR37] Leech J (2017). Potentiality. Analysis.

[CR38] Lewis, D. (1986). Events. In his *Philosophical papers* (Vol. II, pp. 241–69). Oxford: Oxford University Press.

[CR39] Lewis D (1997). Finkish dispositions. Philosophical Quarterly.

[CR40] Livingstone-Banks J (2017). In defence of modal essentialism. Inquiry.

[CR41] Livingstone-Banks, J. (ms.) ‘Essence and possibility’. **(Unpublished manuscript)**

[CR42] Lowe EJ (2008). Two notions of being: Entity and essence. Royal Institute of Philosophy Supplement.

[CR43] Lowe EJ (2012). What is the source of our knowledge of modal truths?. Mind.

[CR44] Lowe EJ, Novak L, Novotny DD, Sousedik P, Svoboda D (2012). Essence and ontology. Metaphysics: Aristotelian, scholastic, analytic.

[CR45] Mackie P (2006). How things might have been: Individuals, kinds, and essential properties.

[CR46] Manley D, Wasserman R (2007). A gradable approach to dispositions. Philosophical Quarterly.

[CR47] Manley D, Wasserman R (2008). On linking dispositions and conditionals. Mind.

[CR48] Marcus RB (1967). Essentialism in modal logic. Nous.

[CR49] McLeod SK (2008). How to reconcile essence with contingent existence. Ratio.

[CR50] McKitrick J (2003). The bare metaphysical possibility of bare dispositions. Philosophy and Phenomenological Research.

[CR51] Michels, R. (*ms*). On how (not) to define modality in terms of essence. **(Unpublished manuscript)**

[CR52] Mumford S (2006). The ungrounded argument. Synthese.

[CR53] Mumford S, Anjum RL (2011). Getting causes from powers.

[CR54] Mumford S, Anjum RL, Gethmann CF (2011). Dispositional modality. Lebeswelt un Wissenschaft: Destsches Jahrbuch für Philosophie 2.

[CR55] Mumford S, Anjum RL, Hüntelmann R, Hattler J (2014). The irreducibility of dispositionality. New scholasticism meets analytic philosophy.

[CR56] Newlands S (2013). Leibniz and the ground of possibility. Philosophical Review.

[CR57] Oderberg D (2007). Real essentialism.

[CR58] Plantinga A (1974). The nature of necessity.

[CR59] Rohrbaugh G, deRossett L (2006). Prevention, independence, and origin. Mind.

[CR60] Rosen, G. (2010). Metaphysical dependence: Grounding and reduction. In B. Hale & A. Hoffmann (Eds.), *Modality: Metaphysics, logic, and epistemology* (pp. 109–136). Oxford: Oxford University Press.

[CR61] Salmon N (1981). Reference and essence.

[CR62] Shalkowski SA (2008). Essence and being. Royal Institute of Philosophy Supplement.

[CR63] Skiles A (2015). Essence in abundance. Canadian Journal of Philosophy.

[CR64] Spencer J (2017). Able to do the impossible. Mind.

[CR65] Steinberg J (2010). Dispositions and subjunctives. Philosophical Studies.

[CR66] Steward S (2015). Ya shouldn’ta couldn’ta wouldn’ta. Synthese.

[CR67] Tahko T (2015). An introduction to metametaphysics.

[CR68] Teitel, T. (2017). Contingent existence and the reduction of modality to essence. *Mind*. 10.1093/mind/fzx001.

[CR69] Torza A (2015). Speaking of essence. Philosophical Quarterly.

[CR70] Turner J (2010). Possibility, by Michael Jubien. Analysis.

[CR71] Vetter B (2011). On linking dispositions and which conditionals?. Mind.

[CR72] Vetter B (2013). Can’ without possible worlds. Semantics for anti-humeans. Philosophers’ Imprint.

[CR73] Vetter B (2014). Dispositions without conditionals. Mind.

[CR74] Vetter B (2015). Potentiality.

[CR75] Wildman N (2013). Modality, sparsity, and essence. Philosophical Quarterly.

[CR76] Wildman N, Jago M (2016). How to be a modalist about essence. Reality making.

[CR77] Wildman, N. (ms–a). Never forget your roots: Re-evaluating the case for origin essentialism. **(Unpublished manuscript)**

[CR78] Wildman, N. (ms–b). What’s wrong with weak necessity? **(Unpublished manuscript)**

[CR79] Wiggins D, Evans G, McDowell J (1976). The de re must: A note on the logical form of essentialist claims. Truth and meaning: Essays in semantics.

[CR80] Zalta EN (2006). Essence and modality. Mind.

